# AI in conflict zones: the potential to revitalise healthcare in Syria and beyond

**DOI:** 10.1136/bmjgh-2024-015755

**Published:** 2024-08-07

**Authors:** Munzer Alkhalil, Aula Abbara, Caroline Grangier, Abdulkarim Ekzayez

**Affiliations:** 1 Research for Health System Strengthening in northern Syria (R4HSSS), Union for Medical and Relief Organizations, Gaziantep, Turkey; 2 LSE IDEAS Conflict and Civicness Research Group, The London School of Economics and Political Science, London, UK; 3 Department of Infection, Imperial College London, London, UK; 4 Syria Public Health Network, London, UK; 5 ESSEC Business School, La Défense, France; 6 Antei Global, Paris, France; 7 War Studies (Research for Health System Strengthening in northern Syria (R4HSSS), King's College London, London, UK; 8 Research & Development, Syria Development Centre, London, UK

**Keywords:** Health policies and all other topics, Global Health, Other diagnostic or tool

**Summary box**

- Artificial intelligence (AI) integration in healthcare, particularly in conflict zones like Syria, has the potential to enhance clinical care, providing remote diagnostics and monitoring capabilities that are crucial in areas with shortages of healthcare workers and specialists.
- Beyond direct clinical care, AI holds the potential to revolutionise healthcare planning, resource allocation, healthcare protection and community health promotion.
- The deployment of AI in healthcare settings, especially in fragile environments, necessitates rigorous ethical considerations and tailored implementation strategies to ensure these technologies support ethical, equitable and effective healthcare delivery without exacerbating existing vulnerabilities or security risks.

## Introduction

 The global health landscape, particularly in conflict-affected areas, faces multiple crises marked by severe healthcare worker (HCWs) shortages and misallocations. For example, in Syria, tens of thousands of HCWs have been forced to leave Syria due to more than a decade of conflict.^1^ A 2019 World Bank and United Nations High Commissioner for Refugees (UNHCR) report noted that from 2010 to 2018, the number of doctors in Syria was reduced from 0.529 to 0.291 per 1000 persons.^2^ This scarcity is even greater in specialised areas such as intensive care units (ICUs), oncology, radiology and laboratory services. In such contexts, leveraging artificial intelligence (AI) becomes not just a beneficial but an essential consideration, particularly in specialities where data-driven decision-making can be harnessed for improved clinical care. In this commentary, we explore the use of AI for direct healthcare services and broader considerations in the humanitarian sector, including potential associated risks.

## Potential areas for AI use in healthcare in conflict zones

### Optimised clinical care

Harnessing AI for clinical care can include its use for diagnosis, monitoring or interventions. The use of AI can enhance existing programmes; for example, soon after the onset of the Syrian conflict after uprisings in March 2011, the Syrian American Medical Society (SAMS), used standard digital technologies including video cameras, digital monitoring and predominantly WhatsApp communication to support remote ward rounds in ICUs in Syria with specialists based in the USA.^3^ This was needed as the casualties induced by intensive shelling increased traumatic injuries, which in turn intensified the demand for, and consequently, the pressure on, the remaining ICU doctors and anaesthetists. Unable to match the need, most of those units were run by technicians^3^ who are trained nurses, anaesthesia technicians and practitioners. This model, while effective, could be significantly enhanced with AI integration as AI tools can offer assistance in early sepsis detection, blood gas analysis,^4^ observations and suggesting adjustments, for example, changes to ventilation settings,^5^ detecting circulatory failure,^6^ managing patient data, protocol guidance and translation services, improving the efficiency and quality of remote medical collaboration. This has not yet been integrated into SAMS ICU projects due to insufficient capacity and risk considerations.

Another example is the use of AI-based sepsis management. A US-based healthcare organisation implemented a rules-based early detection algorithm called Sepsis Prediction and Optimization of Therapy (SPOT) across 173 hospitals, leading to an almost 10% reduction in mortality for patients with severe sepsis in 2019.^7^ ‘SPOT’ is a system that monitors the patients’ investigations, vital signs and all other information continuously to support early detection.^8^ A recent study assessed the impact of a similar deep-learning model called ‘COMPOSER’ for early sepsis prediction on patient outcomes in the UC San Diego Health System, including 6217 adult septic patients between 1 January 2021 and 30 April 2023. The study indicated an absolute reduction in in-hospital sepsis mortality by 1.9% (17% relative decrease) and an absolute increase in sepsis bundle compliance by 5.0% (10% relative increase).^9^

Beyond conflict zones, the COVID-19 pandemic has spotlighted the potential for AI during other healthcare crises. Had AI tools been more widely available during the pandemic peak, several challenges could have been mitigated more effectively. For example, AI could have been crucial in supporting disease detection, evolution and monitoring. A 2020 multicentre study showed that AI algorithms could successfully identify COVID-19 patterns on CT scans with a per-scan sensitivity and specificity for detecting COVID-19 in the independent test set at 90% and 96%, respectively.^10^ AI-based medical imaging techniques have also been very effective for diagnosis due to their cost-effective, wider database and shorter time compared with human based.^11^ Such diagnostic capability could be harnessed for detecting other lung diseases including cancer, chronic obstructive pulmonary disease, interstitial lung disease or tuberculosis.^12^

### Early detection of health-related threats

There has also been increasing interest in using AI in other aspects of the delivery of humanitarian care. This includes detecting and anticipating outbreaks or population movements resulting from conflict or natural disasters. Such examples have been used in UNHCR’s Project Jetson, which used predictive models for forecasting forced displacement after an escalation of violence in Somalia.^13^ AI tracking can be used for logistics or managing healthcare resources, which can often be ineffective in complex humanitarian crises such as Syria. Using AI-driven predictive models could aid in forecasting during outbreaks, which could lead to improved preparedness and response.

### Supporting healthcare planning

Reflecting on the lessons learnt from the COVID-19 response in northwest Syria, a key challenge was the lack of specialised human resources for health and challenges with resource planning and availability.^14^ AI could have supported healthcare planning, allowing the mapping of areas at greatest risk of an outbreak and directing human resources, diagnostics and equipment to these areas. The mismatch between needing equipment and personal protected equipment could also have been mitigated, something which is vital in an area where severe understaffing and the risks to healthcare staff were high. Though models were used to predict the COVID-19 pandemic curve in the area, such models were often high level or inaccurate due to insufficient information about the behaviour of SARS-CoV-2 in such an area.^15^ Harnessing the available information from the COVID-19 pandemic could better inform future anticipatory AI-driven models to improve responses.

### Protection of healthcare

In conflict settings, AI has shown the potential to protect health facilities and workers by predicting airstrikes. One example is the HALA system, which uses AI to predict the likelihood of airstrikes on healthcare facilities.^16^ This system was developed to provide real-time alerts to healthcare providers, allowing them to take necessary precautions. Using AI-powered tools, the system can process and analyse vast amounts of intelligence data in real time. This includes analysing unstructured text in multiple languages, such as Russian, Arabic and English, to provide structured intelligence to decision-makers. Additionally, the system uses advanced situational awareness techniques for conflict management, gathering and fusing data from multiple sources in extremely sensitive environments. Lessons from the HALA system include integrating diverse data sources in different languages, the need for continuous algorithm training and linking knowledge conclusions with timelines to adapt to the evolving nature of conflict for more accurate predictions. These insights could inform future AI-driven models for conflict zones and help mitigate the effects of armed conflict on healthcare delivery.

### Strengthen community healthcare

There was potentially a missed opportunity to capture more advanced AI tools to support community and health education during the COVID-19 pandemic and also the more recent cholera outbreak, which has affected Syria since the summer of 2022. It could be used to integrate and support messaging around health promotion, such as water and hygiene, but also other behaviour change interventions, including healthy lifestyles, smoking cessation, treatment or medication adherence, reduction in substance misuse^17^ and providing mental health support. The latter is becoming increasingly pertinent in Syria where compounding crises (most frequently the February 2023 earthquakes) as well as ongoing attacks have led to a vast crisis in mental health both for the community and also for first responders and health workers.^18^

## Ethical concerns and potential risks of AI in healthcare

However, the use of AI is not without risk. The WHO recently issued new guidance on the ethics and governance of a fast-growing generative AI technology called large multimodal models (LMMs). LMMs can receive one or more types of data input and create diverse outputs that are not limited to the type of data fed into the algorithm. The WHO has identified various risks associated with LMMs in healthcare and systems. In healthcare, there is a risk of inaccurate, incomplete or false responses, poor quality training data, bias, lack of informed consent, manipulation, privacy concerns, reduced clinician–patient interactions, epistemic injustice, delivery of care outside the health system, new burden of learning digital skills and unaccountability for algorithmic content. In health systems, different risks exist, including overestimating AI benefits, unequal AI access, system-wide biases, impacts on labour, dependency on unsuitable AI tools and cybersecurity risks.^19^ Most of the data used for developing AI are from high-resource countries compared with poor data from low-resource, fragile or conflict-affected countries, which generates bias in suggested solutions and a real risk that AI-generated outcomes may also be of poor quality or misleading.^20^ Additionally, the failure of AI tool developers and users to consider contextual factors poses another potential risk, particularly in community healthcare settings. Finally, due to its feed mechanisms, AI cannot detect emerging diseases, new symptoms and manifestations, which threatens to suggest wrong diagnosis.

On the other hand, ethical considerations regarding data privacy and security remain paramount, as they significantly impact patient autonomy, trust in AI and compliance with legal frameworks.^21^ Even where ‘informed’ consent is provided, the person giving it may not know how and where their data are used. Additionally, no clear guidelines for responsibility and accountability in using AI in healthcare are available to address potential errors, harmful outcomes and biases in the predictions generated by AI. These guidelines must define the roles and responsibilities of various stakeholders, such as governments, AI companies, donors, implementing agencies, physicians and healthcare institutions, in cases where misdiagnoses or other patient harm occur.^22^ Finally, the lack of transparency is another concern, possibly due to users’ lack of understanding of the limitations of explainable AI^21^ and knowledge and conflict of interests.

## AI concerns in conflict zones

Medical interventions in conflict areas, including those that are not urgent, may not always be ethically sound and in line with established medical standards. Many reasons for that include weaponised healthcare and data, limited resources, fragmented health systems, weak unions, poor governance and the lack of academic training. Therefore, introducing AI-based interventions into such contexts could exaggerate these issues, given the need for more resources, control and oversight. AI in healthcare requires rigid validation, ethical considerations and regulatory compliance to ensure patient safety and care effectiveness. In environments where basic medical standards are already compromised, AI-based interventions without the necessary infrastructure, expertise and governance frameworks may lead to unintended consequences, including inappropriate treatments and a lack of accountability for outcomes.

In settings of conflict, several challenges to the use of AI to support health and humanitarian care exist, including (1) infrastructure limitations such as limited access to the internet, power Interruptions and a lack of equipment; (2) poor patient data management; (3) fragmented health information systems; (4) lack of training for HCWs, including how to interpret AI outputs in clinical decision-making; (4) ethical implications, including human rights, data privacy, the potential bias of AI algorithms; (5) weak legislative and legal structure and (6) politicisation and weaponisation of healthcare and related data—conflict areas sometimes witness using healthcare and humanitarian aid as punishment tools against some communities so AI can be used to have more control over the health services provision and access and for cybersecurity attacks.

Another challenge which is particularly relevant to conflict areas relates to the ownership of and access to health data in contested areas. In Syria, for example, two epidemiological surveillance systems and three health information systems operate parallelly in different control areas. Information can be weaponised with the potential to affect the ability to translate it into projects or manipulate it to influence conflicting parties’ legitimacy, ability to respond and to attract funding. Moreover, AI can orchestrate sophisticated attacks on health infrastructure, disrupting services and causing chaos. It can generate and disseminate false information about health services, causing panic, mistrust and further destabilisation. AI-based cyberattacks can also be used to target health databases to steal or manipulate personal health records or treatment plans of specific people, leading to harassment, persecution or murder. This can be used strategically to undermine health interventions in certain areas.

It is challenging to determine who is responsible for standardising the use of AI tools and ensuring the safety of patients and the community in conflict zones. Ideally, the government should take on this responsibility, but when the government is a party to the conflict, it falls to local health authorities at the subnational level. For example, in areas outside the control of the Syrian regimen, such as northwest Syria, the emerging ground-up health directorates could take on this task. Furthermore, non-governmental institutions should play an observing role in this regard.

## Conclusion

AI integration in healthcare can potentially enhance clinical care, planning, resource allocation, protection and community healthcare strengthening. However, it is vital to establish clear ethical guidelines and frameworks to govern the use of AI in healthcare in conflict areas, ensuring that these technologies support, rather than undermine, equitable and ethical healthcare services in such settings. This is ideally through ground-up initiatives and dialogue that are contextually feasible so that AI can enhance rather than weaken existing health systems.

## Data Availability

The data supporting this commentary's findings are derived from publicly available sources. The authors did not collect primary data for this work.
